# Polymer Photovoltaic Cells with Rhenium Oxide as Anode Interlayer

**DOI:** 10.1371/journal.pone.0133725

**Published:** 2015-07-30

**Authors:** Jinyu Wei, Dongdong Bai, Liying Yang

**Affiliations:** 1 School of Management, Tianjin University of Technology, Tianjin, China; 2 Key Laboratory of Display Materials & Photoelectric Devices (Ministry of Education), School of Materials Science and Engineering, Tianjin University of Technology, Tianjin, China; Washington State University, UNITED STATES

## Abstract

The effect of a new transition metal oxide, rhenium oxide (ReO_3_), on the performance of polymer solar cells based on regioregular poly(3-hexylthiophene) (P3HT) and methanofullerene [6,6]-phenyl C_61_-butyric acid methyl ester (PCBM) blend as buffer layer was investigated. The effect of the thickness of ReO_3_ layer on electrical characteristics of the polymer solar cells was studied. It is found that insertion of ReO_3_ interfacial layer results in the decreased performance for P3HT: PCBM based solar cells. In order to further explore the mechanism of the decreasing of the open-circuit voltage (V_oc_), the X-ray photoelectron spectroscopy (XPS) is used to investigate the ReO_3_ oxidation states. Kelvin Probe method showed that the work function of the ReO_3_ is estimated to be 5.13eV after thermal evaporation. The results indicated the fact that a portion of ReO_3_ decomposed during thermal evaporation process, resulting in the formation of a buffer layer with a lower work function. As a consequence, a higher energy barrier was generated between the ITO and the active layer.

## Introduction

Organic solar cells are promising candidates for clean energy and have the advantages of flexibility and low production cost, compared to their inorganic counterparts [1–2]. Significant effort has been made toward improving the performance of organic solar cells. The power conversion efficiency (PCE) of polymer solar cells based on a blend of regioregular poly (3-hexylthiophene) (rr-P3HT) and methanofullerene [6,6]-phenyl C_61_-butyric acid methyl ester (PCBM) has reached 5% [3]. Indium tin oxide (ITO) is used as the transparent hole collecting anode. Works on the cell structure modification and insertion of buffer layer material between ITO and the active layer have been reported to improve the short circuit current density (J_sc_) in the polymer solar cell. The efficiency of polymer photovoltaic cells is greatly improved when poly(ethylenedioxythiophene) (PEDOT) doped with poly(styrene sulfonate) (PSS) is used as a buffer layer. The function of PEDOT: PSS is to prevent electron leakage from the bulk heterojunction (BHJ) acceptor to the anode, to enhance photo-generated hole extraction, and to planarize the ITO surface. Nevertheless, recently many researchers have found that PEDOT: PSS also has many disadvantages. For example, they are highly acidic (PH~1) and corrosive to the ITO anode [3]. Spin coated PEDOT: PSS films have large microstructure and electrical inhomogeneities, yielding inconsistent morphologies and electrical conductivity in different regions [4]. These effects may lead to inhomogeneous charge extraction. Furthermore, the using of PEDOT: PSS causes the decreasing of the stability of devices [5–6]. These limitations motivate the development of a more effective interfacial layer to replace the PEDOT: PSS for optimum performance. Previously, T.J. Marks et al have reported a cross-linked blend of poly [9, 9-dioctylfluorene-co-N- [4-(3-methylpropyl)]-diphenylamine] (TFB) +4, 4'-bis [(p-trichlorosilylpropylphenyl) phenylamino] biphenyl (TPDSi_2_) as anode interfacial layer to replace the PEDOT: PSS [7]. Several metal oxides (NiO, V_2_O_5_, MoO_3_) have also been demonstrated as efficient buffer layers in the polymer photovoltaic cells [8–9]. However, their toxicity and high evaporation temperature hinders their practical application in organic electronics. In this work, a PEDOT: PSS/ rhenium oxide (ReO_3_) complex layer was designed and applied as anode buffer layer. ReO_3_ can be evaporated at a lower temperature (~340°C) than MoO_3_ and V_2_O_5_, alleviating the drawbacks of metal oxides in a practical manufacturing process induced by a high evaporation temperature [10]. Moreover, it is said that the ReO_3_ material has a high work function of about 6.0 eV [11]. The performance of polymer bulk heterojunction solar cells is investigated and compared with traditional devices using PEDOT: PSS as a buffer layer.

## Materials and Methods

### Substrate preparation

ITO-coated glass with a sheet resistance of 10 Ω/ square was used for device fabrication. The routine cleaning procedure included sonication in a solution of detergent, deionised (DI) water, and isopropyl alcohol in sequence.

### Device fabrication and characterization

**Fig 1:**
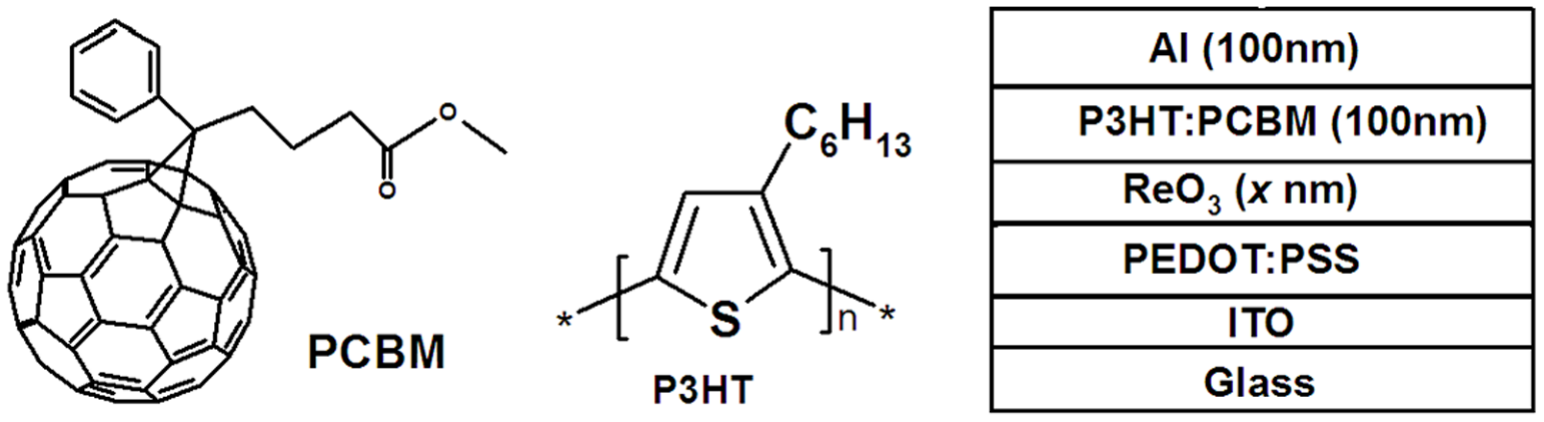
The molecular structure of the materials used in the experiment and the device structure.

The procedure for the solar cell device fabrication is described as follows. The patterned ITO-coated glass was ultra-sonicated with acetone, isopropanol, and de-ionized water for 10 min. The PEDOT: PSS layer of about 25 nm thickness was obtained by spin coating at 3000 rpm for 40 s from an aqueous solution (Baytron P VP Al 4083) on ITO coated glass substrates, followed by baking at 120°C for 5 minutes in air. ReO_3_ (Aldrich, purity 99.9%) was thermally evaporated onto PEDOT:PSS in 0.2, 0.5, 1, and 3 nm thick under a vacuum of about 1×10^−6^ Torr. The blend of P3HT: PCBM (1:0.8 weight-ratio) chlorobenzene solution was spin coated at 1000 rpm for 60 s. The P3HT: PCBM coated substrates were heated at 150°C in a nitrogen atmosphere for 10 min to remove residual solvent before transferring to a vacuum system. Finally, 100nm thickness Al was thermally deposited on top of the active layer under a vacuum of about 1×10^−6^ Torr. A control device using PEDOT: PSS as anode buffer layer is also fabricated for comparsion. The active layer area of the device was 0.09 cm^2^. The un-encapsulated devices were measured in the ambient atmosphere (25±5°C, 35±5% relative humidity) under intensity of 100 mW/cm^2^ white light illuminations by using a 300 W solar simulator (Thermal Oriel 91160) with an AM1.5 G filter. The light intensity was calibrated with an Oriel mono-Si reference cell (CROWNTECH PVM 272 certificated by NREL). I-V curves were swept with a Keithley 2400 source meter from -1 to +1 V in steps of 10 mV with a speed of 0.1 s per step until stable efficiency. The WF of the ITO substrate coated with ReO_3_ interlayer was evaluated by Kelvin probe method in the air using a KP 020 Ambient Kelvin Probe system. The XPS spectra was recorded using a Kratos Axis Ultra DLD spectrometer employing a monochromated Al-K*α* X-ray source (hv = 1486.6 eV), hybrid (magnetic / electrostatic) optics and a multi-channel plate and delay line detector (DLD). All XPS spectra were recorded using an aperture slot of 300 * 700 microns. Survey spectra were recorded with a pass energy of 160 eV, and high resolution spectra with a pass energy of 40 eV. In both wide and narrow scans, the C_1s_ peak at 285.0 eV of adventitious surface hydrocarbons was used to reference charge-induced binding energy shifts in the sample. The molecular structure of the materials used in the experiment and the schematic device structure are shown in Fig 1.

## Results and Discussion

**Fig 2:**
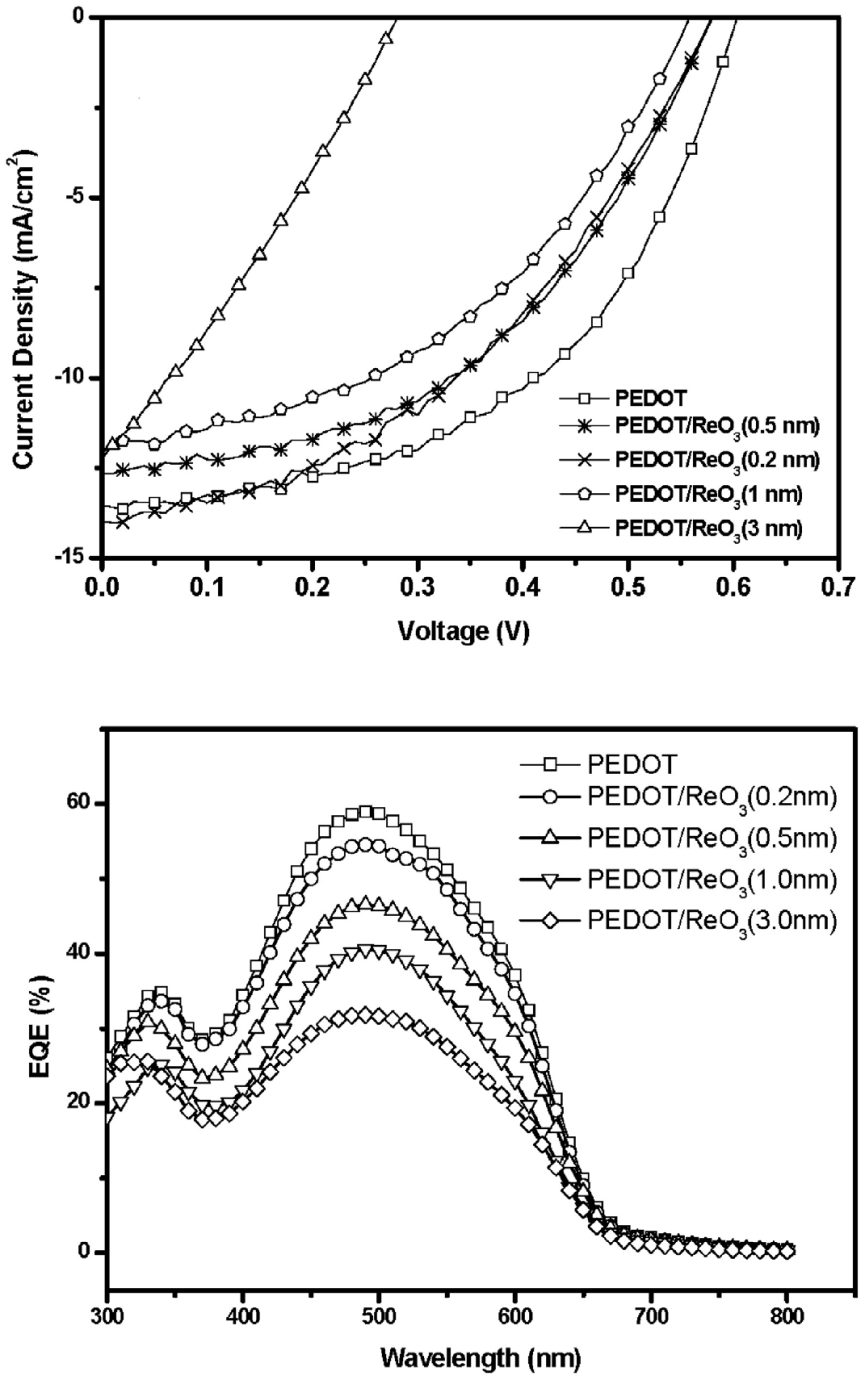
The illuminated J-V characteristics of devices with different ReO3 thickness (a) and IPCE spectra of the devices (b).

**Fig 3:**
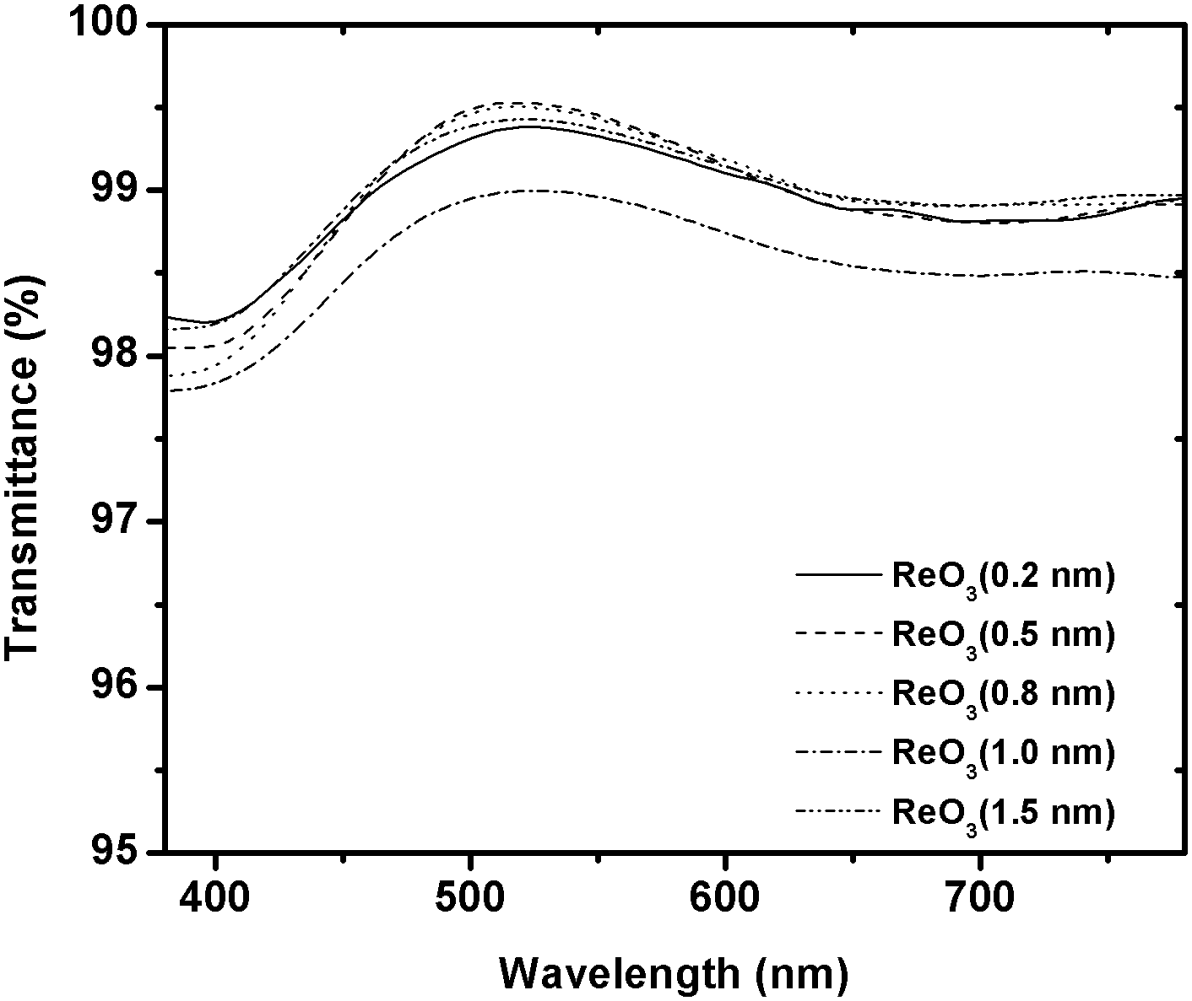
Optical transmittance of ReO3 film with different thickness on quartz substrate.

The J-V characteristics curve for the devices under an intensity of 100 mW/cm^2^ white light illumination with different thickness of ReO_3_ layer is shown in Fig 2. The detailed results are also given in Table 1. The J-V curve for device with ITO/PEDOT: PSS as buffer layer is also shown for comparison. The device with PEDOT: PSS buffer layer provides a significant improvement in the device performance. The PCE is 4.15%, with J_sc_ = 13.54 mA/cm^2^, V_oc_ = 0.60 V, and FF = 51.1%. Furthermore, the insertion a 0.2 nm ReO_3_ layer between PEDOT: PSS and the active layer results in the decrease in J_sc_ of 12.64 mA/cm^2^. The V_oc_ decreases to 0.58V, and FF of 46.4%. The overall PCE is therefore 3.40%. The PCE of the device with 1 nm ReO_3_ is 2.9%, with J_sc_ = 12.04 mA/cm^2^, V_oc_ = 0.55V, and FF = 43.8%. Consequently, the PCE decreases significantly, rising from 4.15% to 2.9%, a 30% decrease compared with the control devices. Fig 2b shows the incident photon-to-current conversion efficiency (IPCE) curves (or the external quantum efficiency (EQE) spectra) of devices with different thickness of ReO_3_ interfacial layer. The IPCE spectra of photovoltaic cells compare very well with those previously reported for P3HT: PCBM blend films. The IPCE results are in agreement with the measured performance. Interestingly, the reported work function of the ReO_3_ and PEDOT: PSS is about 6.0 eV and 5.2 eV, respectively. Theoretically, the performance of the device using ReO_3_ should be better than that of the control device. The observed result is very different from what we have expected. Therefore, we also fabricated devices consisting only ReO_3_ (without PEDOT: PSS) as a buffer layer. The PCE of the optimum device with 10 nm ReO_3_ layer is 2.8%, with J_sc_ = 13.6 mA/cm^2^, V_oc_ = 0.45V, and FF = 53.6%. For organic BHJ solar cells, their maximum value of V_oc max_ depends on the characteristics of the organic/metal contacts. The maximum V_oc_ for the devices with ohmic contacts is governed by the energy difference between the lowest unoccupied molecular orbital (LUMO) of the acceptor and the highest occupied molecular orbital (HOMO) of the donor. On the other hand, in the case of non-ohmic contacts, V_oc max_ is given by the difference in work functions between the anode and the cathode, which follow the metal-insulator-metal model [12]. In our devices, the donor and acceptor materials are the same for all devices. The difference in work functions between the anode (Φ_ITO_ = 4.7eV) and the cathode (Φ_Al_ = 4.28 eV) is 0.42 eV, which is much closer to the measured V_oc_ = 0.45V. To clarify the origin of the V_oc_ decrease in the devices, the optical absorption for ReO_3_ film with different thickness on quartz substrate was measured by an ultraviolet-visible spectrometer. The results are shown in Fig 3. The absorption spectrum is very similar. The average optical transmittance was higher than 98% in the visible range (380-780nm). This small difference could not attribute to more than 30% V_oc_ decrease in our experiment. Therefore, the only possible explanation for the observed phenomena may be caused by reduction in the efficiency of the carrier collection at electrodes. We suspected that a decomposition reaction of ReO_3_ was happened during thermally evaporation, resulting in the formation of a buffer layer with a lower work function. As a consequence, a higher energy barrier was generated between the ITO and the active layer. Kelvin Probe method was used to measure the work function of the ITO substrate coated with PEDOT and ReO_3_, it is revealed that the WF is decrease from 5.20 eV for ITO/PEDOT: PSS to 5.13 eV after thermal evaporation of ReO_3_. Ultraviolet photoelectron spectroscopy (UPS) was also performed to verify the accuracy of the WF of ReO_3_. UPS spectra of ReO_3_ in S1 Fig shows that the WF of ReO_3_ is about 4.94 eV, which is very closely with the results obtained by Kelvin Probe method.

**Table 1:** Parameters for devices with different thickness of ReO_3_.

Anode	Jsc(mA/cm^2^)	Voc(V)	FF(%)	PCE(%)
ITO/PEDOT:PSS (25nm)	13.54	0.60	51.1	4.15
ITO/PEDOT:PSS (25nm)/ ReO_3_ (0.2 nm)	12.64	0.58	46.4	3.40
ITO/PEDOT:PSS (25nm)/ ReO_3_ (0.5 nm)	13.97	0.57	43.0	3.43
ITO/PEDOT:PSS (25nm)/ ReO_3_ (1 nm)	12.04	0.55	43.8	2.90
ITO/PEDOT:PSS (25nm)/ ReO_3_ (3 nm)	12.24	0.28	28.6	0.98

**Fig 4:**
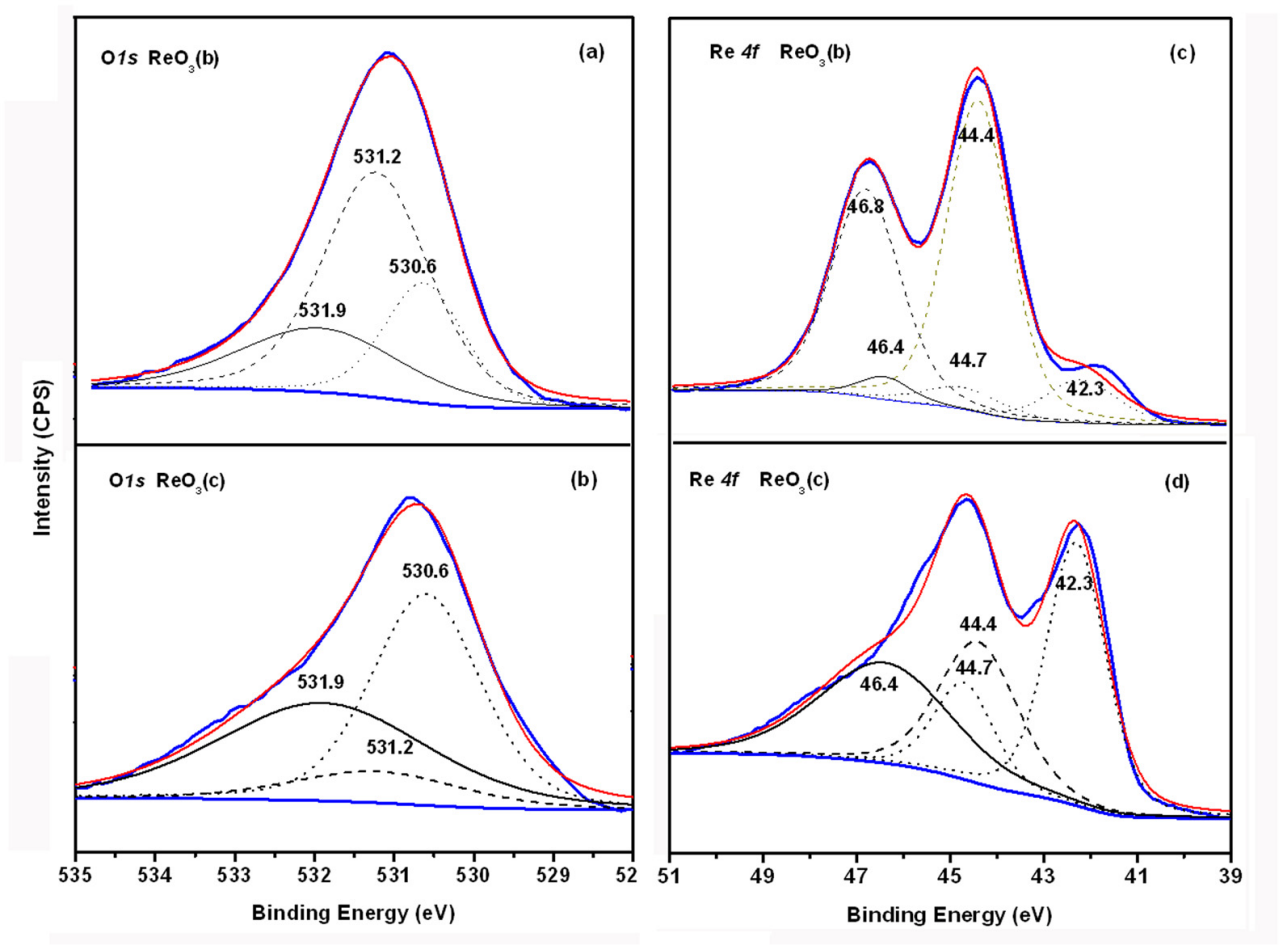
The O1s (a, b) and Re 4f (c, d) core-level spectra region and the peak fitting of the XPS spectra for ReO3 (b) and (c).

XPS measurement offers effective methods to study the electronic at the surface and interfaces of organic electronic devices. The possible decomposition mechanism was further explored by XPS. For simplicity, the original ReO_3_, ReO_3_ deposited on the ITO substrates by thermal evaporation and ReO_3_ remained in the quartz crucible are hereafter to as ReO_3_ (a), ReO_3_ (b), and ReO_3_ (c), respectively. Fig 4 shows the O*1s* and Re *4f* core-level spectra region of the XPS spectra and XPS peak fitting of O_*1s*_ and Re *4f* for ReO_3_ (b) and ReO_3_ (c). The Re *4f* core-level spectrum of the ReO_3_ (b) in Fig 4c shows a main peak at 44.4 eV. Two Re *4f* shoulders peaks can be obviously observed at 42.3 eV and 46.8 eV, respectively. The Re *4f* core-level spectrum of the ReO_3_ (c) in Fig 4d is located at 42.2 and 44.6 eV. More highly resolved (narrow) scans over the Re *4f 7/2*, *5/2* spin-orbit coupling doublet peak (40–52 eV) regions were subsequently acquired. So de-convolution was carried out to deduce oxidation states of Re. De-convolution of the narrow scan profiles for the samples was done using the XPS PEAK program. Shirley (non-linear) baselines, 70% Gaussian/ 30% Lorentzian synthetic peaks were used and a set of constraining criteria applied to provide a measure of objectivity to the deconvolutions. A brief description of the criteria required is as follows: First, the peak areas of the synthetic Re 4f spin-orbit coupling doublets are constrained to be 1.33 (the ratio of the relative degeneracy given by 2J +1 of the 4f 7/2 and 4f 5/2 peaks (where J the spin orbit coupling constant = 7/2 and 5/2). Second, the spin-orbit coupling constants (i.e. the energy difference between the fitted spin orbit coupling doublet Re 4f peaks (ΔEB) are constrained to the literature value of 2.43 eV [13]. Third, the full width at half maximum (FWHM) of the synthetic peak doublets be limited to between 1 and 2 eV. The pick-fitting results in Fig 4c and 4d indicate that some of the ReO_3_ are transformed into ReO_2_ as indicated by the additional spectral line positioned at 42.3 eV and 44.7eV for Re *4f* 7/2 and Re *4f* 5/2, respectively. Spectral line at 44.4 eV and 46.8 eV is assigned to the Re *4f* 7/2 and Re *4f* 5/2 peaks of the ReO_3_ species, respectively. Although no obvious spectral line located at 46.4 eV and 48.8 eV for the existence of for Re^7+^ (Re_2_O_7_) was found in Fig 4c and 4d, we can see the existence of Re_2_O_7_ in Fig 4a and 4b of O_*1s*_ core-level spectra and the peak fitting of the XPS spectra for ReO_3_ (b) and (c).

In comparison with the standard XPS data from NIST database website, deconvolution showed the O_*1s*_ narrow scan to be made up from the contributions of three O_*1s*_ components.[14] This indicated that rhenium oxides was present in at least 3 oxidation states in the sample. The lower binding energy components indicate the presence of Re (IV) oxides located at 530.6 eV while the higher binding energy component, located at 531.2 eV and 531.9 eV respectively, proves the presence of higher oxidation states of Re(VI) and Re(VII). Our study showed that the rhenium oxide (ReO_3_) is not thermally stable. The XPS spectrum shows conclusively that a series of oxidation state Re oxides are existed on the substrates. Thus, we infer that partial thermal evaporated ReO_3_ may tend to decompose into ReO_2_ and Re_2_O_7_ under our experimental conditions following the expected disproportionation thermal decomposition route:

$${\text{ReO}}_{3}\to {\text{ReO}}_{2}{+\text{ Re}}_{2}{\text{O}}_{7}$$

The presence of Re_2_O_7_ in the substrate might be explained due to volatilization.

**Fig 5:**
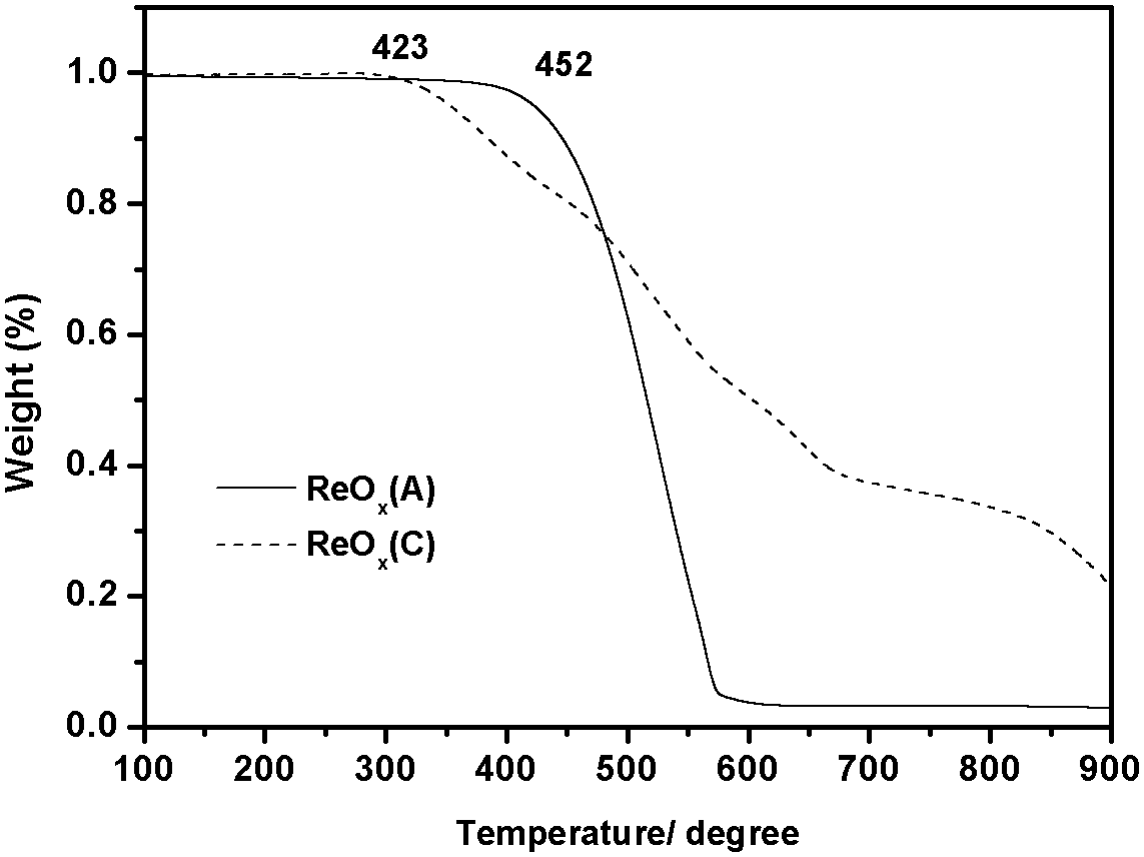
TGA plots of ReO3 (A) and ReO3 (C) with a heating rate of 10°C/min under inert atmosphere.

Thermogravimetric analysis (TGA) is also used to investigate the thermal evaporated ReO_3_. Fig 5 shows the TGA results of ReO_3_ (a) and ReO_3_ (c), respectively. The TGA analysis reveals that under inert atmosphere, the onset decomposition temperature (Td, 5% weight loss) for ReO_3_ (a) and ReO_3_ (c) were 452 and 423°C, respectively. After that rapid weight loss is observed. It implies that some reactions were taken place during thermal evaporation process. All evidences indicated that ReO_3_ with poor thermal stability is not adequate as potential anode buffer layer for the fabrication process of organic photovoltaic devices.

## Conclusion

In conclusion, we have investigated polymer photovoltaic devices based on P3HT: PCBM blend using ReO_3_ as interfacial buffer layer. The effect of thickness of ReO_3_ on electrical characteristics of the device was also studied. A J_sc_ of 13.6 mA/cm^2^, V_oc_ of 0.45 V, and a PCE of 2.8% were obtained for optimum device under simulated AM1.5G 100 mW/cm^2^ intensity in air. Compared with traditional device using PEDOT: PSS as buffer layer, insertion of ReO_3_ interfacial layer result in the decreased performance for P3HT: PCBM solar cells. XPS and Kelvin Probe results revealed that a portion of ReO_3_ decomposed into ReO_2_ during thermal evaporation process, resulting in the formation of a buffer layer with a lower work function. As a result, a higher energy barrier was generated between the ITO and the active layer. Our results also revealed that the WF of the ReO_3_ material might be overestimated.

## Supporting Information

S1 Fig UPS spectra of ReO_3_. (DOC)
