# Supplementary material for: Polymer Photovoltaic Cells with Rhenium Oxide as Anode Interlayer
Source: PLoS One. 2015 Jul 30;10(7):e0133725. doi: 10.1371/journal.pone.0133725 (PMC4520519; doi:10.1371/journal.pone.0133725)
Supplement: S1 Fig — (DOC) [file pone.0133725.s001.doc]

**Supporting Information**

**Polymer photovoltaic cells with rhenium oxide as anode interlayer**

Jinyu Wei^1^, Dongdong Bai^1^, Liying Yang^2*^

**1** School of Management, Tianjin University of Technology, Tianjin, China

**2** Key Laboratory of Display Materials & Photoelectric Devices (Ministry of Education), School of Materials Science and Engineering, Tianjin University of Technology, Tianjin, China

E-mail: weijinyu2010@126.com

**Measurement of the Work function (WF) by Kelvin Probe**

We first measure the WF of the tip of the Kelvin probe system by performing a single-point measurement of the gold sample. The default WF of gold is 5100 meV. The Contact Potential Difference (CPD) measured between the tip and the gold sample is 200 mV. Therefor, the WF of the Tip is 4900 meV. Remove the gold sample from the sample holder and place the ReOx sample. The CPD value of the sample and tip is about 230 mV. So, the WF of the ReOx layer is 5130 meV.

**Measurement of the Work function (WF) by ultraviolet photoelectron spectroscopy (UPS)**

The ultraviolet photoemission spectroscopy (UPS) was used to probe the WF of the ReOx sample. UPS were performed in a Kratos AXIS Ultra-DLD ultrahigh vacuum photoemission spectroscopy system and He I excitations (21.2 eV) were used for radiation source. The energy of a photon is given by the Einstein relation:

E = h ν (1)

To recover from the ionisedstate the atom can emit another photon:

h ν= E _Fermi_- E _Cutoff_+ Ø _Workfunction_ (2)

Ø _Workfunction_ = h ν- E _Fermi_ + E _Cutoff_ (3)

S1 Fig. UPS spectra of ReO_3_

Therefore, Ø _Workfunction_ = h ν- E _Fermi_ + E _Cutoff_

=21.22-21.98+5.70

=4.94 eV
